# Simple Executive Function as an endophenotype of autism-ADHD, and differing associations between simple versus complex Executive Functions and autism/ADHD traits

**DOI:** 10.1038/s41598-025-87863-2

**Published:** 2025-02-10

**Authors:** Alexandra Hendry, Rachael Bedford, Mary Agyapong, Jannath Begum Ali, Tessel Bazelmans, Mutluhan Ersoy, Amy Goodwin, Luke Mason, Nisha Narvekar, Greg Pasco, Mark. H. Johnson, Emily J. H. Jones, Tony Charman, The STAARS Team, Alexandra Hendry, Rachael Bedford, Mary Agyapong, Jannath Begum Ali, Tessel Bazelmans, Mutluhan Ersoy, Amy Goodwin, Luke Mason, Nisha Narvekar, Greg Pasco, Mark. H. Johnson, Emily J. H. Jones, Tony Charman, Leila Dafner, Laurel Fish, Teodora Gliga, Rianne Haartsen, Hanna Halkola, Rebecca Holman, Sarah Kalwarowsky, Anna Kolesnik, Chloë Taylor

**Affiliations:** 1 Department of Experimental Psychology, University of Oxford, Oxford, UK; 2 Centre for Brain and Behaviour, Department of Psychology, School of Biological and Behavioural Sciences, Queen Mary University of London, London, UK; 3 Psychology Department, Institute of Psychiatry, Psychology & Neuroscience, King’s College London, London, UK; 4 Centre for Brain and Cognitive Development, Birkbeck College, University of London, London, UK; 5 Department of Psychology, Kastamonu University, Kastamonu, Turkey; 6 Department of Forensic and Neurodevelopmental Sciences, Institute of Psychiatry, Psychology and Neuroscience, King’s College, London, UK; 7 Department of Psychology, University of Cambridge, Cambridge, UK; 8 Department of Child and Adolescent Psychiatry and the MRC Centre for Developmental Neurobiology, Institute of Psychiatry, Psychology & Neuroscience, King’s College London, London, UK; 9 School of Psychology, University of East Anglia, Norwich, UK

**Keywords:** Executive Function, Autism, ADHD, Endophenotype, Toddler, Preschool, Human behaviour, Cognitive control

## Abstract

Autism and ADHD are associated with difficulties with Executive Functions (EFs), but the prevalence and nature of these difficulties in early development is not well understood. In this longitudinal study, 107 children with a family history of autism and/or ADHD (FH-autism/ADHD), and 24 children with No-FH-autism/ADHD completed multiple EF tasks (5 at age 2 years, 7 at age 3 years). Parents reported on their child’s autism- (Q-CHAT at age 2, SRS-2 at age 3), and ADHD-related traits (CBCL DSM-ADHD scale, both ages). Compared to the No-FH-autism/ADHD group, the FH-autism/ADHD group showed lower scores on simple EFs (involving response inhibition, and holding in mind) at ages 2 and 3. Exploratory analysis linked FH-autism specifically with lower Executive Attention (top-down attentional control) at age 2, and the combination of FH-autism and FH-ADHD with lower Complex EF (involving selectively deploying responses, or updating information) at age 3. Three-year-olds’ Simple EF scores were negatively associated with ADHD-related traits. Complex EF scores were negatively associated with autism traits (before correcting for multiple comparisons). Toddlers with a family history of autism and/or ADHD may benefit from interventions to support simple EF development, whilst those already showing autistic traits may benefit from support with more-complex EF skills.

## Introduction

Executive Functions (EFs) are the cognitive tools that enable us to control attention and behaviour in pursuit of a goal. In school-aged children and adults, EFs are generally considered to include some combination of inhibitory control, working memory or updating, and cognitive flexibility or shifting^1–3^. These skills are important for planning, problem-solving and adapting to changing circumstances, and require internally-directed cognitive control. EFs develop within the first 3 years of life, building from skills such as inhibiting a response, and holding in mind a set of rules to guide behaviour^4–6^ (referred to as ‘simple EF’s in Garon, Bryson and Smith’s^6^ hierarchical model of EF development), and top-down attentional control (‘executive attention’)^4^. From these develop the ability to coordinate and selectively deploy responses, and to update and manipulate information (referred to as ‘complex EF’ skills by Garon et al.^6^).

Autism and ADHD are among the most common forms of neurodevelopmental differences, and are highly co-occurent^7,8^ and heritable^9–11^. Autism and ADHD have both been linked to EF difficulties, with both overlap and divergence in terms of the specific sub-constructs affected. Older children and adults with autism and ADHD often show some difficulties with inhibitory control, working memory and planning, whilst cognitive flexibility difficulties are generally observed only in the autistic group^12–16^. Amongst preschoolers specifically, autistic children are most often characterized as having weaker cognitive flexibility and inhibition skills, whereas children with ADHD are most often characterized as having weaker inhibition, planning, and working memory skills^17^. Within these broad trends, it is also the case however that children with autism and/or ADHD show high heterogeneity in EF ability^18,19^.

When EF difficulties are observed amongst children and adults with autism or ADHD, they have been linked to lower quality of life and general daily life skills^20–24^, and are predictive of more severe socioemotional problems such as anxiety or depression^25–27^. Understanding more about the emergence and prevalence of EF difficulties in children with, or at increased likelihood of, autism and/or ADHD is key to discerning how, when and to whom we should provide support to limit negative outcomes for neurodivergent individuals.

One influential theory is that EF is a transdiagnostic endophenotype linking autism and ADHD^28^. By endophenotype we mean a measurable trait of genetic etiology that isheritable, sits at an intermediate position between clinical symptoms and their underpinning neurobiological mechanisms, and presents at higher rates within affected families than the general population^29^. Support for this theory comes from studies showing that siblings of individuals with a diagnosis of autism or ADHD tend to show EF difficulties themselves^30–38^– although others have reported no group differences between children with and without a family history of autism when children with clinical levels of autistic traits themselves are excluded^39,40^. To date, there is limited research into when EF difficulties emerge in children with a family history of autism or ADHD, and which aspects of EF are most affected in early development. One exception is St John et al.^41^who found that children with a family history of autism showed comparable performance to those with no family history of autism on reversal trials of the A-not-B task (considered to index inhibitory control) at 12 months, but lower scores at age 24 months. No group differences were found with regards to overall accuracy (considered to index working memory). Lower reversal scores were most pronounced for the sub-set of infants with a family history of autism who showed clinical levels of autism traits at 24 months^41^. No published studies have yet examined performance across a broad battery of simple and more-complex EF measures amongst children aged 3 years or younger with a family history of autism or ADHD.

In this study, we examine whether EF difficulties are an early-emerging transdiagnostic endophenotype of autism and / or ADHD. We first test whether a family history of autism and/or ADHD is linked to lower EF scores at ages 2 and 3 years. We then explore whether there are specific differences between children with no family history of autism or ADHD, and those with a family history of autism only, ADHD only, or both autism and ADHD. We next test whether associations between early EFs and autistic and ADHD traits are evident at age 2 and/or 3 years, and explore what longitudinal associations (between 2 and 3 years) may tell us about likely developmental pathways.

## Methods

### Participants

Participants were enrolled in the BASIS study at age 5 or 10 months, and seen again at ages 14 months, 2 years and 3 years; only participants who were seen for a 2- (*n* = 126) and/or 3-year (*n* = 124) visit are reported here. The Family History of autism or ADHD (FH-autism/ADHD) sample were enrolled if they had a first-degree relative (most commonly sibling, but sometimes parent) with a community clinical diagnosis of autism or ADHD, or a first-degree relative with elevated ADHD traits (who were then screened using a short version of one the Conners suite of measures); see Begum Ali et al.^42^ for a detailed elaboration of the cohort ascertainment approach. The No-FH-autism/ADHD sample were enrolled if they had a typically-developing sibling and no first-degree relative with autism or ADHD. To enable us to test for specific effects of family history of autism, or of ADHD, and their interaction, each participant was coded for FH-autism (where ‘1’ indicates one or more first-degree relatives is autistic, and ‘0’ indicates no known presence of autism in a parent or sibling) and FH-ADHD (where ‘1’ indicates one or more first-degree relatives has ADHD, and ‘0’ indicates no known presence of ADHD in a parent or sibling).

Both samples were required to have a gestational age > 36 weeks, and no known medical or developmental condition at the point of enrolment. Informed written consent was provided by parents. Ethical approval was granted by the National Research Ethics Service (13/LO/0751) and the Research Ethics Committee, Department of Psychological Sciences, Birkbeck, University of London. All methods were performed in accordance with the Declaration of Helskinki. Participant demographics are presented in Table 1.

**Table 1:** Participant demographic characteristics.

	No-FH-autism/ ADHD	FH-autism/ADHD			Significant group differences
		FH-autism-only	FH-ADHD-only	FH-autism-and-ADHD	
Mean age in months at 2-year visit (SD)	25.04 (1.19)	25.41 (1.54)	24.96 (0.79)	25.27 (1.27)	None^1^
Mean age in months at 3-year visit (SD)	37.35 (1.86)	37.64 (1.46)	37.15 (1.13)	37.79 (2.79)	None^1^
*N*	24	71	20	16	
Male *N* (%) Females *N* (%)	13 (54) 11 (46)	36 (51) 35 (49)	11 (55) 9 (45)	11 (69) 5 (31)	None^2^
Maternal ethnicity: White/European/Irish (%) Asian (%) African/African-Caribbean/Mixed heritage (%)	86 9 5	82 7 10	93 7 0	93 0 7	None^2^
Highest maternal education level: Further education^3^(%) Undergraduate degree^3^(%) Postgraduate degree^3^(%)	9 35 57	27 50 24	60 27 13	30 35 35	FH autism/ADHD < No FH autism/ADHD^1^ FH autism + ADHD < No FH autism/ADHD^1^

^1^based on 2-tailed t-tests of FH autism/ADHD versus no-FH autism/ADHD, and univariate ANOVAs testing for the effect of FH-autism, FH-ADHD and FH-autism+ADHD.

^2^based on Chi-Sq tests for FH group and sub-group contrasts.

^3^or equivalent.

### Executive Function measures

At ages 2 and 3 years, during the course of a full-day visit that included behavioural assessments of cognitive and socio-emotional functioning, autism traits and temperament, not described here, participants were presented with a battery of tasks designed to elicit EF. These tasks are summarized in Table 2, with further elaboration and details of data quality checks and exclusions in SM1.1.

**Table 2:** Behavioural measures of EF.

Task (and task type)	Target construct	Summary description (see SM 1.1 for further details)	Dependent variable
***Antisaccade***^43,44^ (eyetracking)	Attentional inhibitory control	Indexes inhibitory aspects of attentional control in terms of suppression of automatic saccades to a distractor and execution of an anticipatory saccade in the opposite direction	Proportion of valid block 2 trials where the infant made a saccade to the target location not preceded by a prosaccade to the distractor
***Reversal Learning***^45,46^ (eyetracking)	Attentional shifting	Captures ability to control attention according to a new rule, following a switch from a previous opposing rule	Number of correct anticipations during the reversal block as a proportion of all valid reversal block trials
***Hidden Toy***^45^ (eyetracking)	Updating	Indexes the ability to hold in mind an object’s location when occluded from view, and update that representation with new information over the course of multiple hiding events	Proportion of locations correctly fixated as a proportion of the number of valid trials
***Prohibition***^47,48^ (object-based)	Directed global inhibition	Captures ability/willingness to comply with a prohibition to touch an appealing toy	Latency to touch the prohibited toy (seconds)
***Go/No-Go***^49^ (touchscreen)	Competitive inhibition (block 1), Set-shifting (block 2)	Block 1: Indexes ability to follow a rule to produce a response on ‘Go’ trials (75%) and inhibit that response on ‘No-go’ trials (25%). Block 2: Captures ability to control responses according to a new rule as the stimuli for ‘Go’ and ‘No-go’ trials are reversed	d prime (d′) sensitivity index: a standardized difference between the hit rate (proportion of go trials to which there was a correct tap) and the false alarm rate (proportion of no-go trials to which there was an incorrect tap)
Hide-and-Seek (touchscreen)	Set-shifting	Captures ability to respond according to changing rules by searching first by 1 dimension (Block 1: tap the big house); then another (Block 2: tap the red house); then by another rule within the same dimension (Block 3: tap the yellow house)	Number of correct trials, as a proportion of all valid trials
***Delayed Alternation***^50,51^ (touchscreen)	Working Memory	Indexes the ability to follow an alternating rule (tap the right side, then the left, then the right and so on) in order to access a reward (cartoon, which introduces a 7 s delay between responses)	Number of correct retrievals as a percentage of the total trials administered
***Spin the Pots***^52^ (object based)	Updating	Captures ability to update a mental representation of the location of hidden objects across multiple trials: 6 treasures are placed in 1 of 8 visually distinct pots. Participants search 1 pot per trial, with the pots covered and spun between trials	The number of errors made, subtracted from 12 (the maximum number of searches)

Different subsets of the battery were administered at the two-year (Antisaccade, Reversal Learning, Hidden Toy, Prohibition, Delayed Alternation) and three-year visit (Antisaccade, Reversal Learning, Delayed Alternation, Go/No-Go, Hide-and-Seek, Prohibition, Spin the Pots) in order to minimize ceiling and floor effects. Tasks were presented in a fixed order as detailed in SM1.1, designed to maximize engagement with each task. The parent was present in the room for all tasks but was instructed to not prompt the child at any point, and was out of direct eye-line of the child.

#### Data reduction: Analytic approach

To minimize the number of statistical tests we aimed to first reduce the behavioural data at each timepoint. The 2-year data did not meet criteria for data reduction via Exploratory Factor Analysis (EFA) as Bartlett’s Test of Sphericity was not significant (χ^2^(10) = 9.863, *p* = 0.453); therefore data were reduced via mean composite score only for the variables that were significantly associated. For the 3-year data, Bartlett’s Test of Sphericity χ^2^(28) = 77.144, *p* < 0.001) was significant and Kaiser–Meyer–Olkin MSA = 0.553 which is just acceptable for EFA (Kaiser, 1974), therefore data were reduced via EFA.

#### Data reduction: 2-year data

Performance on the Antisaccade and Reversal Learning task was significantly correlated at age 2 (see Table 3): these scores were therefore combined into a composite variable – which we titled Executive Attention – by computing the mean of the two standardised variables (*z*scores). None of the other measures were significantly correlated at age 2, and were therefore considered separately. According to Garon’s et al.’s hierarchical model of EF^6^, these remaining measures involve either simple response inhibition (Prohibition) or holding information in mind over a delay (Hidden Toy, Delayed Alternation), and can be characterized as simple EF tasks.

**Table 3:** Cross-sectional associations between EF task performance scores at the 2-year visit.

	Antisaccade	Reversal Learning	Hidden Toy	Prohibition
Reversal Learning	.258*			
Hidden Toy	.055	.116		
Prohibition	.010 (.033)	-.059 (.033)	.066 (.043)	
Delayed Alternation	.038	-.054	-.096	-.019 (-.035)

Zero-order Pearson correlations shown by default. Spearman Rho correlations shown in parenthesis.

*p < .05.

#### Data reduction: 3-year data

As shown in Table 4, at 3 years, between-task correlations were modest, but scores for each task were significantly correlated with at least one other task. EFA indicated that the 3-year data could be reduced to 2 factors; see SM 2.3. The tasks loading most strongly onto Factor 1 involve either simple response inhibition (Prohibition), or holding information in mind over a delay (Delayed Alternation); as above, these can be characterized as simple EF tasks. Performance on the Hide-and-Seek task also loaded onto Factor 1. Previously, it has been shown that performance on this kind of shifting task is linked to the ability to maintain task set (a ‘simple EF’), and to shift to a different task set (a ‘complex EF’), and that most 3-year-olds perform below chance with regards to shifting set. In our dataset, average overall accuracy scores for both groups was well above chance (see Supplementary Table 2.3.1), indicating that the shifting demands of the task were relatively low. We therefore interpret the Hide-and-Seek loadings onto Factor 1 as indexing maintaining task set (and the lower loadings onto Factor 2 as indexing shifting). Taking all of the above into account, we labelled Factor 1 Simple EF.

**Table 4:** Cross-sectional associations between EF task performance scores at the 3-year visit.

	Anti-saccade	Reversal	Prohibition	G/NG 1	G/NG 2	H&S	DA
Reversal Learning	.300*						
Prohibition	.161	-.134 (-.117)					
Go/No-Go block 1	.160	.231	.198 (.227*)				
Go/No-Go block 2	.079	.196	.090 (.064)	.176			
Hide-and-Seek	.023	.055	.023 (.174)	.100	.352*		
Delayed Alternation	.080	-.089	.406** (.288*)	.134	.127	.273*	
Spin the Pots	.188	.127	.050 (.119)	.185	.281*	.038	.090

Zero-order Pearson correlations shown by default. Spearman Rho correlations shown in parenthesis.

**p < .01,*p < .05.

The tasks loading onto Factor 2 involved complex response inhibition (Go/No go), shifting responses in accordance with a new task set (Go/No go block 2, Reversal learning), updating information within a complex working memory task (Spin the pots) and inhibiting a prepotent response (Antisaccade), which can all be considered complex EFs. We note that Garon et al.^6^ characterized Antisaccade as a Simple response inhibition task but argue that as it involves inhibition of a cued response *and* activation of a sub-dominant response (making a saccade to the contralateral side) it is better characterized as a complex response inhibition task. Taking all of the above into account, we labelled Factor 2 Complex EF.

Descriptive statistics for the summary EF scores and for the clinical data, are presented in Table 5. Descriptive statistics for the task-level EF scores are presented in Supplementary Table 2.1.1.

**Table 5:** Descriptive statistics, by Family History group and tests of FH group differences.

		**No-FH-autism/ADHD**	**FH-autism/ADHD**	**Test statistic**	***p***	**Effect size (d)**
**2 years**						
Executive Attention	Mean (SD)	.21 (.61)	-.06 (.92)	*t* = 1.598	.059	.310
	*n*	20	86			
Hidden Toy	Mean (SD)	.52 (.20)	.47 (.18)	*t* = 1.039	.170	.255
	*n*	21	79			
Prohibition	Mean (SD)	20.76 (12.45)	13.82 (13.50)	*U* = 587.500	.015^a^	.438
	*n*	21	79			
Delayed alternation	Mean (SD)	.39 (.17)	.41 (.22)	*U* = 870.000	.338	.082
	*n*	22	84			
Q-CHAT raw score	Mean (SD)	19.92 (5.66)	25.34 (11.00)	*t* = −3.219	.001	-.528
	*n*	21	93			
CBCL-ADHD raw score	Mean (SD)	3.48 (2.36)	4.69 (3.09)	*U* = 825.500	.062	.286
	*n*	22	95			
**3 years**						
Simple EF	Mean (SD)	.25 (.61)	-.06 (1.05)	*t* = 1.794	.040	.308
	*n*	20	103			
Complex EF	Mean (SD)	-.01 (.71)	.00 (1.05)	*t* = -.055	.478	.013
	*n*	20	103			
SRS-2 raw score	Mean (SD)	23.37 (9.46)	41.46 (32.03)	*U* = 520.000	.007	.488
	*n*	19	85			
CBCL-ADHD raw score	Mean (SD)	3.05 (2.16)	4.37 (3.27)	*U* = 761.000	.073	.277
	*n*	21	91			

^a^ Remains significant at *p* < .05 after excluding children scoring in the clinical range for autism or ADHD at age 3 (n = 16).

### Autism and ADHD trait measures

Parent report of autistic traits was collected via the Q-CHAT^53^ at the 2-year visit, and the Social Responsiveness Scale 2-Preschool Form (SRS-2)^54^ at the 3-year visit. The Q-CHAT is a 25-item questionnaire designed to detect early autism-related behaviours at 18–24 months^53^. The Q-Chat uses a 5-level Likert response format from 0 (“*never*”) to 4 (“*many times a day*”) to yield a continuous measure of autistic traits on a score range of 0–100. The SRS-2 comprises 65 items relating to social awareness, social cognition, social communication, social motivation and restricted interests and repetitive behaviour. It uses a 4-point scale from 0 (“*not true*”) to 3 (“*almost always true*”) to yield a continuous measure of autistic traits on a score range of 0–195.

Emerging ADHD traits were measured at both visits using the parent-reported ADHD DSM-oriented subscale of the Child Behaviour Checklist-Preschool (CBCL-P 1.5–5)^55^. This subscale comprises 6 statements that assess a child’s inattentive and hyperactive behaviour (e.g., “Can’t concentrate, can’t pay attention for long”, “Can’t sit still, restless, or hyperactive”). Parents are asked to indicate how well each statement described their child’s behaviour as observed within the past 2 months on a 3-point Likert scale from 0 (“*not true*”) to 2 (“*very true or often true*”) to yield a continuous measure of ADHD-related traits on a score range of 0–12.

### Analytic approach

One-tailed hypothesis-driven t-tests (or the Mann–Whitney *U* test for non-normally distributed Prohibition and Delayed alternation data) were used to test the hypothesis that the FH-autism/ADHD group (comprising children with a family history of autism only, ADHD only, or both autism and ADHD) would show lower EF scores compared with the No-FH-autism/ADHD group. Additionally, one-tailed hypothesis-driven t-tests (or the Mann–Whitney *U* test for non-normally distributed CBCL and SRS-2 data) were used to test if the FH-autism/ADHD group showed evidence of the Broader Autism Phenotype (i.e. elevated autism traits) or Broader ADHD phenotype (i.e. elevated ADHD traits). Alpha was set at 0.05 for all tests.

We then explored the effect of specific family history by running univariate ANOVAS with FH-autism, FH-ADHD as fixed factors within a full factorial model. To check whether group effects may be driven by a clinical sub-group, in secondary analyses we excluded children who scored above recommended clinical thresholds for autism (SRS-2 raw scores > 60) or ADHD (CBCL-ADHD raw scores > 10) at age 3 (*n* = 18, 14 of whom scored above threshold for autism, one of whom scored above threshold for autism and ADHD, and three of whom additionally above threshold for ADHD only).

To test for cross-sectional associations between EF task performance and autism and ADHD traits at ages 2 and 3, we used correlation analysis (Spearman’s *R* due to high skew in the clinical variables), applying the Benjamini–Hochberg procedure to correct for multiple comparisons. Linear regression was used to test for longitudinal associations between 2 year EF scores and 3- year autism and ADHD traits (visual inspection confirmed that the residuals were normally distributed), with trait scores at age 2 included as a predictor in follow-up analyses to gain insight into mechanistic pathways.

There were no significant group or sub-group differences by child sex, and behavioural scores did not significantly differ by sex; see SM 2.2 for details. Maternal education differed by family history group but was not associated with any of the EF variables (as tested by linear regression). Therefore no additional covariates were included in the analyses.

## Results

### FH group comparisons

**Fig. 1:**
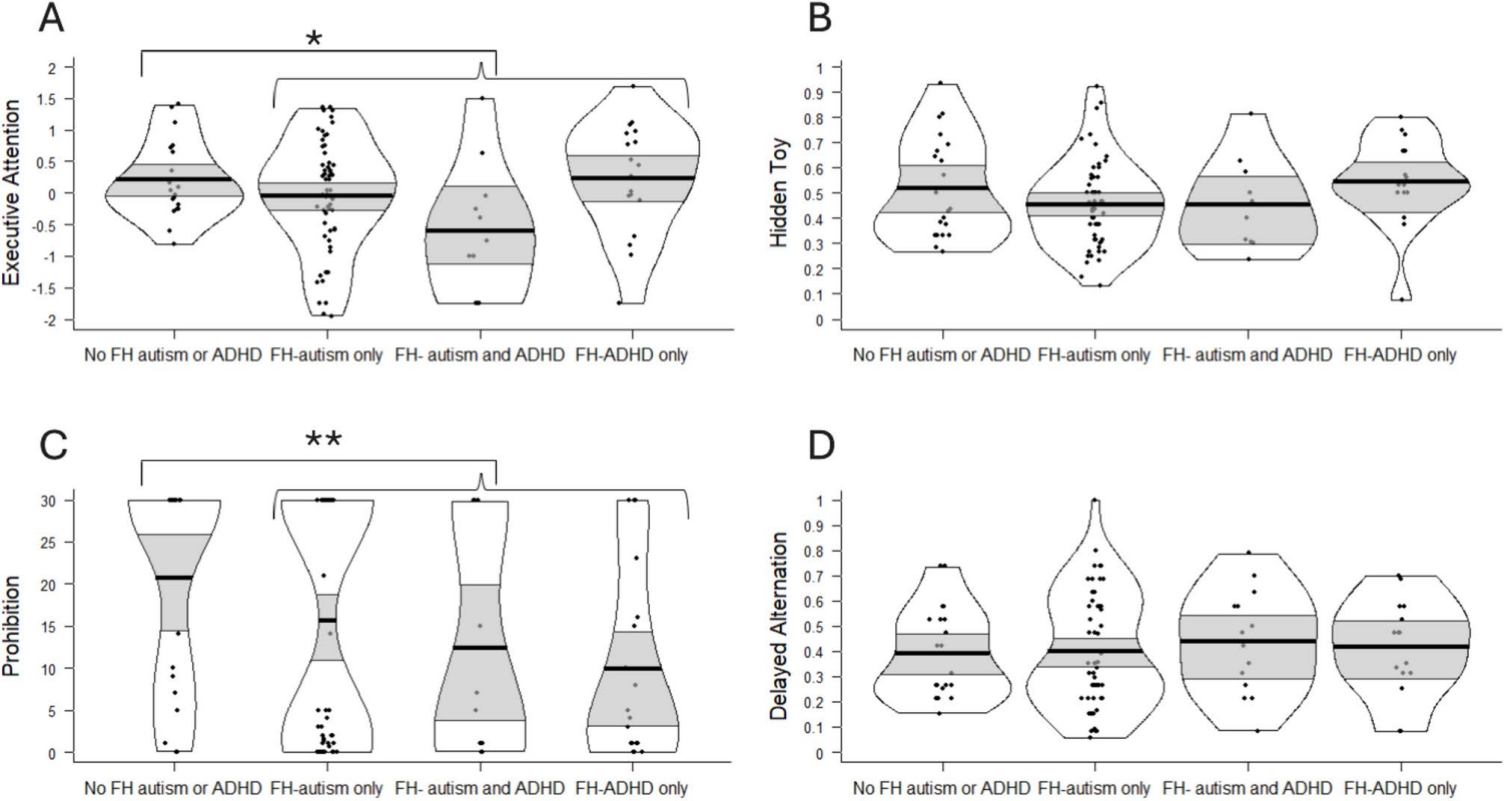
Behavioural performance scores by Family History (FH) at age 2 years. The bold black line indicates the group mean, the light grey bands the 95% confidence interval, and the black border the full data distribution. Note that data is presented in FH subgroups for descriptive purposes only. Primary analyses contrasting the ‘No-FH-autism/ADHD’ group with the ‘FH-autism only’, ‘FH-autism and ADHD’ and ‘FH-ADHD only’ sub-groups combined (‘FH-autism/ADHD’) showed group mean differences for Executive Attention (**A**) (*p < .10) and Prohibition (**C**) (**p < .05).

At age 2, the FH-autism/ADHD group showed significantly lower Prohibition scores compared with their peers, and a non-significant trend towards lower Executive Attention scores; see Table 5 and Fig. 1. When the Executive Attention tasks were considered individually, FH group contrasts were not significant for Antisaccade (*p* = 0.154) or Reversal Learning (*p* = 0.073).

**Fig. 2:**
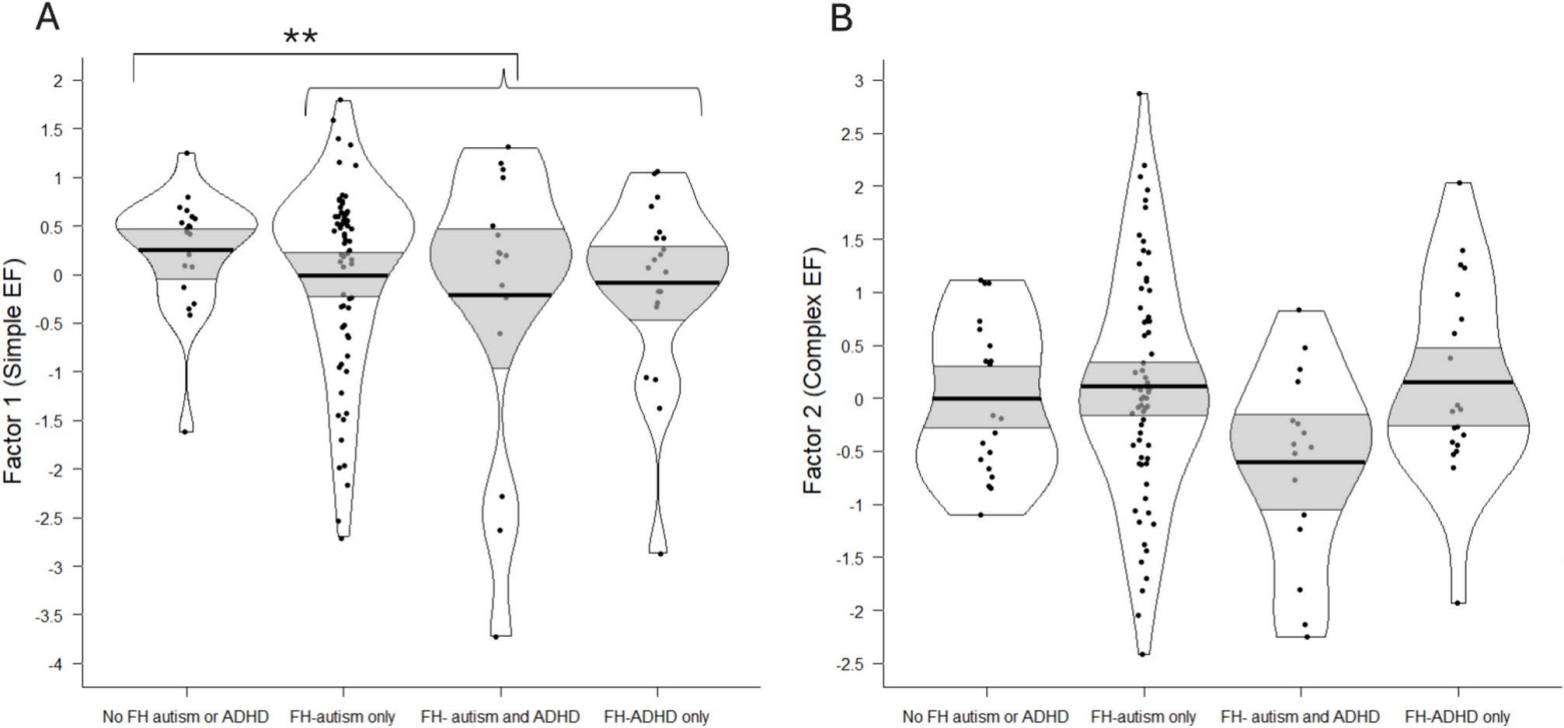
Behavioural performance scores by Family History (FH) group at age 3 years. The bold black line indicates the group mean, the light grey bands the 95% confidence interval, and the black border the full data distribution. Note that data is presented in FH subgroups for descriptive purposes only. Primary analyses contrasting the ‘No-FH-autism/ADHD’ group with the ‘FH-autism only’, ‘FH-autism and ADHD’ and ‘FH-ADHD only’ sub-groups combined (‘FH-autism/ADHD’) showed group mean differences for Simple EF (**A**) (**p < .05).

At age 3, the FH-autism/ADHD group showed lower Simple EF scores; see Table 5 and Fig. 2.

When children scoring in the clinical range for autism or ADHD at age 3 were excluded, only the group difference on 2-year Prohibition scores remained significant, but the FH-autism/ADHD group showed a non-significant trend towards lower Simple EF scores (*t*(50.71) = 1.55, *p* = 0.063, *d* = 0.358).

Exploratory analyses of specific family history effects at age 2 indicated that FH-autism was associated with significantly lower Executive Attention scores; see Table 6. When the Executive Attention tasks were considered individually, FH-autism was associated with significantly lower Antisaccade scores (*F*(1,96) = 5.45, *p* = 0.022) but the effect of FH-autism on Reversal Learning was not significant (*F*(1,74) = 3.289, *p* = 0.074). FH-ADHD was associated with significantly lower Prohibition scores; see Table 6. As the residuals for the Prohibition ANOVA were not normally distributed, we verified with Mann–Whitney tests that there was a significant effect of FH-ADHD (*p* = 0.021) but not FH-autism (*p* = 0.327) on Prohibition scores.

**Table 6:** Specific family history effects on EF performance (based on univariate ANOVAS with FH-autism, FH-ADHD as fixed factors within a full factorial model).

2-year visit	*F* for full univariate ANOVA Model	*F* for effect of FH-autism	*F* for effect of FH-ADHD	*F* for interaction effect of FH-autism and FH- ADHD
Executive Attention	2.696*	7.569** ^a^	1.796	2.088
Hidden Toy	1.356	3.102	0.082	0.098
Prohibition	2.387	0.191	5.436*^b^	1.627
Delayed alternation	0.183	0.111	0.475	0.021
**3-year visit**				
Simple EF	0.698	0.803	1.618	0.016
Complex EF	2.448†	2.389	1.792	4.435*^a^

***p < .001, **p < .01, *p < .05 †*p* < .10.

^a^ Remains significant after excluding children scoring in the clinical range for autism or ADHD at age 3 (*n* = 14 for Executive Attention *n* = 18 for Complex EF).

^b^ Not significant after excluding children scoring in the clinical range for autism or ADHD at age 3 (n = 16).

At age 3, exploratory analyses indicated no significant main effects of FH-autism or FH-ADHD on Simple or Complex EF, but rather an interaction effect of FH-autism and FH-ADHD on Complex EF, whereby children with a family history of both autism and ADHD showed the lowest scores (see Table 6 and Fig. 2B).

### Cross-sectional associations between EF task performance and autism and ADHD traits

At age 2, latency to touch on the Prohibition task was negatively associated with autism traits (r_s_ = −0.303, *p* = 0.004) and ADHD traits (r_s_ = −0.260, *p* = 0.013). No other 2-year EF performance scores were significantly associated with concurrent autism or ADHD traits, and correlation coefficients were all < 0.2.

At age 3, Simple EF factor scores were significantly negatively associated with ADHD traits (r_s_ = −0.248, *p* = 0.009). Complex EF factor scores were negatively associated with autism traits (r_s_ = −0.204, *p* = 0.039) but this did not survive correction for multiple comparisons. No other 3-year EF factor scores were significantly associated with concurrent autism or ADHD traits, and correlation coefficients were all < 0.2.

### Longitudinal associations between EF task performance and autism and ADHD traits

Shorter latency to touch on the Prohibition task at age 2 years was predictive of higher autistic traits at age 3 years (β = −0.324, *p* = 0.004, *r*^2^ = 0.105). However, when autistic traits at 2 years were included in the model the overall regression was statistically significant (*r*^2^ = 0.566, *F*(2,71) = 45.06, *p* < 0.001), autistic traits at age 2 significantly predicted autistic traits at age 3 (β = 2.093, *p* < 0.001), but Prohibition scores at age 2 did not significantly predict autistic traits at age 3 (β = −0.032, *p* = 0.566).

Shorter latency to touch on the Prohibition task at age 2 years was also predictive of higher ADHD traits at 3 years (β = −0.395, *p* < 0.001, *r*^2^ = 0.155). When ADHD traits at 2 years were included in the model the overall regression was statistically significant (*r*^2^ = 0.640, *F*(2,79) = 68.44, *p* < 0.001), ADHD traits at age 2 significantly predicted ADHD traits at age 3 (β = 0.798, *p* < 0.001), and Prohibition scores at age 2 showed a trend-level predictive association to ADHD traits at age 3 (β = 0.035, *p* = 0.050).No other predictive associations from EF performance at 2 years to 3-year autism and ADHD traits were found.

High autistic traits at 2 years were predictive of lower Simple EF scores at 3 years (β = −0.277, *p* = 0.004, *r*^2^ = 0.077), but not Complex EF scores (β = −0.172, *p* = 0.077, *r*^2^ = 0.029). High ADHD traits at 2 years had no significant predictive association to either Simple EF (β = −0.154, *p* = 0.109, *r*^2^ = 0.024) or Complex EF scores at 3 years (β = −0.137, *p* = 0.154, *r*^2^ = 0.019).

## Discussion

In this longitudinal study of children with and without a family history of autism and ADHD we tested the hypothesis that a family history of autism, or ADHD, or both is linked to lower Executive Function (EF) scores at ages 2 and 3 years, and investigated concurrent and longitudinal associations between EFs and autistic and ADHD traits.

### Simple EF is an endophenotype of autism and ADHD

We first considered family history of autism and ADHD in broad, transdiagnostic, terms, by combining children with FH-autism, or FH-ADHD, or FH-autism-and ADHD, in one group (FH-autism/ADHD). Compared with those with no family history of autism or ADHD (no-FH-autism/ADHD), toddlers with a FH-autism/ADHD tended to show lower scores for simple EFs (i.e. relating to the ability to inhibit a response, or hold information in mind). Specifically, at age 2 years the FH-autism/ADHD group showed lower average scores on a Prohibition task (but no significant differences on our other two simple EF tasks), and at 3 years lower average scores on a latent factor that we labelled Simple EF because it was loaded on by tasks with demands relating to maintenance of task set, holding information in mind over a delay, or simple response inhibition^6^. When participants who scored above clinical thresholds on parent-reported autism or ADHD traits at age 3 years were excluded, only 2-year Prohibition scores remained significantly different between groups, but the 3-year effect was at trend with a consistent direction and effect size, so may have become non-significant simply due to loss in power. We therefore conclude that two- and three-year-olds with a family history of autism/ADHD are more likely to show lower scores on (some) measures of Simple EF, even if they do not meet clinical criteria for autism or ADHD themselves. Exploratory analysis indicated that differences in Prohibition scores at age 2 were associated particularly with FH-ADHD. There were no specific FH sub-group effects on Simple EF at age 3, but we note that contrasts had slightly less than 80% power to detect a moderate effect. Previous work has reported that, compared with peers with no family history of autism, 2-year-olds with a family history of autism, but who do not meet clinical cut-offs for autism themselves have lower parent-reported inhibitory control scores (relating to the ability to exercise simple response inhibition)^56,57^. Our results add to this literature by providing evidence of FH-autism/ADHD differences using experimental measures of control of behaviour. In sum, these results are consistent with the argument that EF is a transdiagnostic endophenotype of autism or ADHD^28^, at least with regards to simple EF.

### Executive Attention may be an endophenotype of autism (but not ADHD)

At two years, we also observed that the FH-autism/ADHD group showed a non-significant trend towards lower Executive Attention scores (as indexed by performance on two eyetracking tasks indexing attentional control, neither of which showed a significant FH-autism/ADHD group difference when considered individually). Follow-up exploratory sub-group analysis indicated that FH-autism specifically is associated with lower Executive Attention scores, even after excluding participants who scored above clinical thresholds on parent-reported autism or ADHD traits at age 3 years, and that this effect was driven by Antisaccade performance. This finding is consistent with previous eyetracking studies indicating that infants with a family history of autism show differences in executive attention development between 9 and 15 months^58^, yet contrasts with follow-up research linking the experimental measure of attention to genetic liability for ADHD rather than autism^59^. Although the FH-autism and FH-ADHD contrasts were similarly powered, it is possible that small FH-ADHD effects were missed due to lack of power. Additionally, as our findings were based on performance on just two tasks, which only showed weak-to-moderate associations at the 2-year visit and which loaded onto a broader factor (discussed below) at the 3-year-visit, further investigation with a broader battery of tasks is required before drawing any firm conclusions as to whether executive attention in toddlerhood constitutes a distinct endophenotype of autism.

### Difficulties with complex EF are evident only for three-year-olds with a family history of both autism and ADHD

Contrary to our hypothesis, three-year-olds with a family history of autism/ADHD did not score lower with regards to complex EF (indexing the ability to engage high-order cognitive control skills with regards to competitive inhibition, updating, working memory and shifting^6^). In fact, as shown in Fig. 2, many in the FH-autism group, and some in the FH-ADHD group, showed high Complex EF factor scores. Previous work has highlighted the high heterogeneity in EFs amongst children with a clinical diagnosis of autism or ADHD^18,19^ – we enhance this literature to show that such heterogeneity extends to young children with a family history of autism/ADHD, with some children showing pronounced difficulties and others showing superior skills to any in the No-FH-autism/ADHD group. Nevertheless, exploratory analysis indicated that one subgroup did show, on average, significantly lower Complex EF scores; three-year-olds with a family history of autism *and*ADHD. Given that complex EFs at preschool age are proposed to build from earlier executive attention and simple EF skills^4^, it is plausible that lower complex EF performance amongst three-year-olds with a family history of autism and ADHD is a downstream consequence of exposure to both genetic propensity for lower simple EF (proposed above as an endophenotype of autism and ADHD, and particularly evident for the FH-ADHD group at age 2), and genetic propensity for lower executive attention (proposed above as a possible endophenotype of autism). This interpretation suggests how heterogeneity in complex EF is nevertheless compatible with the idea of EF(s) as an endophenotype, by taking into account the potential interactive effects of genetic liabilities and neural systems^60^.

### Autism- and ADHD-related traits are associated with dissociable EF profiles by age 3

Whilst simple EF appears to be negatively associated with both autism and ADHD at the trait level at age 2 (in terms of significant cross-sectional correlations with Prohibition performance), by age 3 our results indicate some dissociable associations between simple and complex EF and autism- versus ADHD-related traits. A recent review summarized that, based on task-level performance and parent report, autistic preschoolers are most often characterized as having weaker cognitive flexibility and inhibition skills, whereas preschoolers with ADHD are most often characterized as having weaker inhibition, planning, and working memory skills^17^. By applying exploratory factor analysis to a large battery of tasks we have been able to take a data-driven approach to this question and found that three-year-olds’ Simple EF factor scores were significantly negatively associated with ADHD-related traits but not autism traits, whilst Complex EF factor scores were negatively associated only with autism traits (although this result did not survive correction for multiple comparisons). Further, by combining a prospective longitudinal design with parent report of ADHD-related traits we have been able to circumvent the difficulty that ADHD is not commonly diagnosed before the age of 5 years^61^, and collect data on EF-ADHD trait associations as young as 2 years. Whilst this is a strength of the study, we also acknowledge that there is potential for measurement noise within parent report measures, particularly at this young age, and that ADHD (and autism) traits might not be clearly expressed as early as age 3. Indeed, ADHD traits were only elevated at the trend level for the FH-autism/ADHD group at both ages 2 and 3 years. There would therefore be considerable value in conducting further assessments of the study sample at an age when ADHD may be more robustly clinically assessed, in order to test for EF differences amongst groups meeting clinical criteria for autism and/or ADHD; such work is already underway.

We cannot be certain why we only found dissociable associations between EF and autism-related versus ADHD-related traits at age 3 and not age 2. It may be that autism-specific associations with complex EF skills are only apparent from age 3 because many of these skills only start influencing behaviour in a meaningful way around that age. Indeed, because piloting with 2-year-olds revealed floor effects on many of the tasks that, at age 3 years, loaded onto the Complex EF factor those tasks were not included in our 2-year battery. However, future studies may be able to take advantage of developments in new complex EF tasks suitable for younger toddlers^48,62^ to present a more-comprehensive battery.

### EFs and autism and ADHD traits are entwined from their first emergence

Longitudinal analysis indicated bi-directional associations between simple EF and autism and ADHD traits: High autistic traits at 2 years were predictive of lower Simple EF factor scores at 3 years, whilst Prohibition task performance at age 2 years was predictive of autistic and ADHD-related traits at age 3 years. Of note, the predictive association from Prohibition scores to ADHD-related traits remained even after controlling for ADHD traits at age 2. An important caveat is that Prohibition scores accounted for only 16% of the variance in ADHD scores, and were based on a single trial. As is widely observed for this task^63,64^, Prohibition scores had a largely bimodal distribution (toddlers tended to either touch the prohibited object within a few seconds or wait the full 30 s for the prohibition to be lifted); this may have constrained the predictive utility of analyses involving this task. These results, however, provide behavioral evidence consistent with research using parent report indicating that simple EF plays a role in the manifestation of both ADHD and autistic traits^65–69^. Our results do not indicate that difficulties with complex EF are secondary to neurodivergent traits as neither autism nor ADHD traits at age 2 were predictive of 3-year complex EF scores.

### Translational implications

Our results indicate that children with a family history of autism and/or ADHD may benefit from support in the development of simple EF skills, whilst children already showing autistic traits may additionally benefit from opportunities to practise more-complex EF skills from early in development. However, it is important not to assume that low EF scores in toddlerhood are inherently detrimental. Recent work has shown that 3-year-olds with low parent-reported simple EFs are more-efficient problem-solvers, and that low scores on the same prohibition task used here was associated with higher generativity during problem solving amongst 2-year-olds^63^. Therefore, interventions should balance support for EF development with opportunities to build on existing strengths, and be careful not to inadvertently send the inaccurate message that children with a family history of autism/ADHD, or already showing autistic traits, will struggle in all aspects of their life. Indeed, further research is needed to investigate the implication of early differences in EF for cognitive development, skill acquisition and mental wellbeing amongst children with a family history of autism or ADHD.

## Conclusions

This study measured performance across a range of EF tasks from children with and without a family history of autism and/or ADHD, at ages 2 and 3 years. Our relatively large battery of tasks enabled us to discern possible areas of strength and difficulty. In particular we were able to corroborate reports (at a younger age than previously) that simple EF is an endophenotype of autism and ADHD. Exploratory findings, that merit further investigation, indicate that executive attention may be an endophenotype of autism (but not ADHD), whilst difficulties with complex EF are evident only for three-year-olds with a family history of both autism and ADHD.

We also found that autism- and ADHD-related traits are associated with dissociable EF profiles by age 3; whereby Simple EF factor scores were significantly negatively associated with ADHD-related traits but not autism traits, whilst Complex EF factor scores were negatively associated only with autism traits (although this result did not survive correction for multiple comparisons). Additionally, our longitudinal design enabled us to identify that autism and ADHD traits are reciprocally linked with EF across development from as early as age 2 years.

## Supplementary Information

Supplementary Information.

## Data Availability

Data and research materials supporting the results in the article are stored in the British Autism Study of Infant Siblings (BASIS) Network Data Repository and are subject to the BASIS data sharing policies https://www.basisnetwork.org
