# Supplementary material for: Simple Executive Function as an endophenotype of autism-ADHD, and differing associations between simple versus complex Executive Functions and autism/ADHD traits
Source: Sci Rep. 2025 Feb 10;15:4925. doi: 10.1038/s41598-025-87863-2 (PMC11811128; doi:10.1038/s41598-025-87863-2)
Supplement: Supplementary file 1 — Supplementary Information. [file 41598_2025_87863_MOESM1_ESM.docx]

# Supplementary Materials 1: Further methods detail

## 1.1 EF measures

The order of presentation of the behavioral tasks at the 2-year visit was as follows:

1. Antisaccade, Reversal Learning, Hidden Toy (blocks interspersed with each other)
2. Prohibition
3. Delayed Alternation

The order of presentation of the behavioral tasks at the 3-year visit was as follows:

1. Spin the Pots
2. Antisaccade, Reversal Learning (blocks interspersed with each other)
3. Delayed Alternation
4. Go/No-Go
5. Hide-and-Seek
6. Prohibition

The majority of participants provided data during a visit to [LAB BLINDED FOR PEER REVIEW]. Parents of 1 participant at the 2-year visit (FH-autism), and 2 participants at the 3-year visit (both FH-autism) requested a home visit for pragmatic or personal reasons; eyetracking and touchscreen tasks were not included in the protocol for these visits. Data collection for the data included in this study began in April 2015 and was completed in November 2019.

Supplementary Table 1.1. Data quality checks and exclusions for each EF measure

| **Task** | Inclusion/exclusion category | *N:* 2-year-olds | *N:* 3-year-olds |
| --- | --- | --- | --- |
| Antisaccade | Valid data | 101 | 87 |
|  | Insufficient valid trials | 16 | 30 |
|  | Did not engage with eyetracking tasks | 7 | 3 |
|  | No visit | 6 | 8 |
|  | Not administered (home visit) | 1 | 2 |
|  | Technical error | 1 | 2 |
| Reversal Learning | Valid data | 89 | 98 |
|  | Insufficient valid trials in reversal phase | 37 | 16 |
|  | Did not engage with eyetracking tasks | 7 | 3 |
|  | Did not pass learning phase | 2 | 1 |
|  | No visit | 6 | 8 |
|  | Not administered (home visit) | 1 | 2 |
|  | Technical error | 0 | 4 |
| Hidden Toy | Valid data | 101 |  |
|  | Insufficient valid trials | 16 |  |
|  | Did not engage with eyetracking tasks | 7 |  |
|  | No visit | 6 |  |
|  | Not administered (home visit) | 1 |  |
|  | Technical error | 1 |  |
| Prohibition | Valid data | 100 | 120 |
|  | Not in protocol at time of visit | 20 |  |
|  | Did not engage with task | 4 | 3 |
|  | Missing video | 2 | 1 |
|  | No visit | 6 | 8 |
| Go/No-Go | Valid data |  | 80 |
|  | Insufficient valid practice trials |  | 1 |
|  | Insufficient valid trials in block 1 |  | 22 |
|  | Indiscriminate responding |  | 3 |
|  | Non-responsiveness |  | 5 |
|  | Child refused |  | 2 |
|  | <75% trials correctly registered in block 1 |  | 2 |
|  | Data not recorded |  | 9 |
|  | No visit |  | 8 |
|  | Not administered (no time) |  | 1 |
|  | Not administered (home visit) |  | 2 |
| Hide-and-Seek | Valid data – pre-switch |  | 81 |
|  | Valid data – post-switch |  | 70 |
|  | Did not demonstrate understanding of size/colour in the concept-checking phase |  | 27 |
|  | Insufficient correct trials in block 1 (pre-switch); scored less than 75% |  | 11 |
|  | Child refused |  | 6 |
|  | Parental interference |  | 1 |
|  | >2 trials mis-registered in switch block |  | 1 |
|  | Data not recorded |  | 5 |
|  | No visit |  | 8 |
|  | Not administered (no time) |  | 1 |
|  | Not administered (home visit) |  | 2 |
| Delayed Alternation | Valid data | 106 | 107 |
|  | Child quit within <11 trials | 13 | 2 |
|  | Admin error | 2 | 1 |
|  | Data not recorded | 0 | 5 |
|  | >2 trials mis-registered | 4 | 6 |
|  | No visit | 6 | 8 |
|  | Not administered (no time) | 0 | 1 |
|  | Not administered (home visit) | 1 | 2 |
| Spin the Pots | Valid data |  | 110 |
|  | Child quit before completing |  | 1 |
|  | Child not attending during task set up |  | 1 |
|  | Data not recorded |  | 4 |
|  | No visit |  | 8 |
|  | Not administered (no time) |  | 6 |
|  | Not administered (home visit) |  | 1 |

## 1.2 Eyetracking tasks

Eyetracking data was acquired from a Tobii TX-300 (Tobii AB, Sweden) at a sampling rate of 120Hz, with children sitting approximately 60cm from the 23” screen (58.42cm x 28.6cm, 52.0° x 26.8° @ 60cm, native resolution of 1920 x 1080 pixels and an aspect ratio of 16:9).

At the start of the eyetracking assessment the experimenter positioned each participant in front of the eyetracker; most often on their parent’s lap, but independently if preferred. Online feedback was displayed during this initial stage, allowing the researcher to adjust the child’s position to be chosen as close as possible to the centre of the eye tracker head box, to maximise data quality. An automatic five-point calibration was then performed. All tasks began when the participant fixated a gaze-contingent central fixation stimulus, at a size of 3cm x 3cm (2.86° x 2.86° at 60cm viewing distance).

Stimuli were presented on Apple Macbook Pro computers, using custom-written stimulus presentation framework (Task Engine, sites.google.com/site/taskenginedoc/), running in Matlab using Psychtoolbox 3 (Brainard, 1997; Kleiner et al., 2007) and the GStreamer library (gstreamer.freedesktop.org) for video decoding. Raw eye tracking data was acquired via the Tobii Gaze Analytics SDK 3.0, processed and saved to disk. Trial onset and offset was associated with the current sample of gaze data, and time-stamped in the eye tracker’s time format. When a video was playing, an additional timestamp was recorded every 30 frames, in order to ensure constant synchronisation between stimuli and data.

Tasks were administered in blocks that were intermixed with each other and with other tasks not included here (total eyetracking battery duration approx. 40 minutes). If the participant became bored or fussy, the experimenter could skip the current trial and move on to the next. Skipped trials were marked in the data and excluded from analysis.

### Antisaccade

The Antisaccade task aims to index inhibitory aspects of attentional control in terms of suppression of automatic saccades to a distractor and execution of an anticipatory saccade in the opposite direction (Johnson, 1995; Portugal, Bedford, Cheung, Mason, & Smith, 2021; Scerif et al., 2005). Each trial started with the presentation of a central animation (a star, subtending 3°×3°). When this central stimulus was fixated, a distractor (a black circle, subtending 3°×3° with 17° to the right or left of the screen) appeared for 200ms, then 1000ms after the distractor disappeared a target stimulus (a red circle, subtending 4°×4° with 17° eccentricity) was presented in the contralateral location. When this target was fixated, an attractive animation of an animal with a sound replaced the target. If the participant looked at the target side before its presentation, the animation started immediately. Thus, the participant is implicitly trained to inhibit their response to the distractor in order to more quickly fixate the reward. Within participant, the distractor and target did not change sides across trials but side was balanced across participants and within FH groups.

Location of looks and reaction times to stimuli were measured offline. In each trial, it was determined (1) whether the participant looked at the distractor and (2) whether they looked at the target location before or up to 100 ms post target onset (= anticipatory look). All trials were automatically validated based on gaze quality flags; see Braithwaite et al. (2023) for details. If during a trial the participant did not look to the distractor nor the target location before target appearance the trial was excluded on the basis that the participant failed to orient to the distractor. Only valid trials were considered for further computation of measures.

The task was presented in two blocks, each comprising 12 trials, with other eyetracking tasks interspersed between the blocks to maintain engagement. As the initial trials can be seen as training trials, the dependent variable was proportion of valid trials in the second block which were characterized as antisaccades (i.e. the participant made a saccade to the target location, which was not preceded by a prosaccade to the distractor). Participants with fewer than 6 valid trials were excluded; see Supplementary Table 1.1.

### Reversal Learning

The Reversal Learning task aims to capture infants’ ability to control their attention according to a new rule, following a switch from a previous opposing rule (Braithwaite et al., 2023; Kovacs & Mehler, 2009; Wass, Porayska-Pomsta, & Johnson, 2011). In each trial two purple rectangles 17cm by 13cm (16.1° x 12.5° @ 60cm) were presented on the left and right of the screen (1.5cm, or 1.43° from the outermost edge) for 2000ms or until one of the rectangles was fixated by the participant (whichever was soonest). At this point, the rectangle was replaced by a video of the same dimensions, showing a 2s clip of an engaging animation (Thomas the Tank Engine). After the clip had played, the next trial began. Areas of Interest were placed around the location of each of the rectangles (within one of which the video played) and dilated by 2° to account for poor calibration.

The side fixated in the first trial was recorded, but if no side was fixated after 2000ms, the side of video presentation was determined randomly. In the following eight trials (“learning block”), the video was presented on the opposite side to that of the first trial. This learning block ended after three anticipatory saccades were made to the correct side of the screen, or eight further trials had been presented (whichever was soonest). In the subsequent reversal block the rewarded side was reversed, for nine trials. The first reversal block trial was not scored as this served to indicate the new rule). The dependent variable was the number of correct anticipations during the reversal block as a proportion of all valid reversal block trials.

Trials were considered invalid if Saccadic Reaction Time was less than 300ms or if no anticipatory saccade was made. Participants were excluded from analysis if they made fewer than 2 anticipatory saccades (regardless of correctness). Data were excluded in each phase if participants completed fewer than 2 valid trials for that phase, or if they did not ‘pass’ the learning phase by making 3 or more anticipatory looks to the initially-rewarded location; see Supplementary Table 1.1.

### Hidden Toy

The Hidden Toy task aims to index the ability to hold in mind an object’s location when occluded from view, and to update that representation with a new location over the course of multiple hiding events (Braithwaite et al., 2023). A representation of two theatre stages with a lowered curtain were presented on either side of the screen (16.0cm x 23.4cm, 15.2° x 22.1° @ 60cm). In each trial, a toy appeared in the top centre of the screen then dropped to the vertical centre of the screen (over 500ms). Once the participant fixated the toy, the curtains on both stages lifted (over 400ms). The toy then moved (over 750ms) to one of the stages – chosen at random – remained motionless for 200ms, then span for 400ms to engage attention. Both stage curtains then lowered (over 400ms), hiding the toy. A central stimulus was then presented to draw the participant’s attention back to the centre of the screen. Upon fixation, it paused for 200ms, span for 200ms, then disappeared. Once the participant fixated one of the two stage curtains for a minimum of 100ms, the chosen curtain was raised (over 400ms), revealing either the toy (if this was the stage that the toy had moved to earlier in the trial) or an empty stage. If revealed, the toy spun for 400ms as a reward then disappeared via the bottom of the screen. The curtain then lowered and the next trial began. The dependent variable was the proportion of curtains correctly fixated as a proportion of the number of valid trials. Trials were considered invalid and were discarded if 2000ms elapsed without either curtain being fixated. Data were excluded if participants completed fewer than 10 valid trials; see Table 2.

## 1.3 Touchscreen Tasks

Touchscreen tasks were administered on a touchscreen monitor (Iiyama ProLite T1634MC-B3X) connected to a Dell PC (Intel Core i3-3220 CPU @ 3.30GHz 3.29 GHz, with 4 GB RAM and a 32-bit Operating system) running Windows 7 Professional. Tasks were coded and run in EPrime vn 2.0 (Professional). The participant was seated at a low table with the experimenter sitting next to them. Prior to the experimental tasks described below, participants engaged in a short warm-up game involving tapping coloured bubbles on the touchscreen. The purpose of this was to familiarise the participant with the touchscreen and to screen for difficulties in tapping a target, of which none were observed.

### Go/No-Go

This novel version of the classic Go/No-Go paradigm (Howard & Okely, 2015), involved presentation of a static stimulus – a cartoon cat or dog – for 1500ms, or until the screen was tapped. Participants were required to tap the screen on “go” trials (“Splat the cat”, to trigger a coloured splodge appearing on the cat, with a squelching sound effect) and not tap the screen on “no-go” trials (“Don’t splat the dog”; if no tap was given, a gentle dog yapping sound was played; if tapped the trial ended with no other stimulus change). The majority of stimuli were go trials: this generates a prepotent tendency to respond, requiring participants to inhibit this response on no-go trials. Prior to commencing, participants were given instructions with a demonstration (4 trials; 3 go, 1 no-go, then given the opportunity to practice (4 trials; 3 go, 1 no-go) before the instructions were recapped. In block 1 of the task there were 24 trials (75% go trials). Participants were then told that they would now play a different game and the aim of the new game was to “Splat the dog and don’t splat the cat”. The researcher then initiated block 2 of the task, comprising 24 trials (75% go trials). Stimuli were presented in pseudo-random order, such that a block never begins with a no-go stimulus and no more than two successive trials are no-go stimuli.

To ensure only valid responses were included in inhibitory control scores, data was screened using the session recording to identify potential grounds for exclusion of individual data points. Individual trials were marked as invalid if the following conditions were met:

- participant non-engagement; the participant was not looking at the screen, or was looking but was not within reach of the touchscreen for at least 50% of the trial duration (*n*trials block 1=339, *n*trials block 2=528)
- response time < 200ms (because responding was likely to have been initiated before rather than in response to the stimulus) (*n*trials block 1=63, *n*trials block 2=65)
- experimenter interference; they touched the screen during an experimental trial (by error, or because they needed to clean the screen) (*n*trials block 1=7, *n*trials block 2 =20).

Additionally, as per Howard and Melhuish (2017), data were excluded (from both blocks) on the basis of:

- indiscriminant responding if Block 1 go accuracy exceeded 80% and no-go accuracy fell below 20%; (*n*=3). Additionally, data were excluded from block 2 only if block 2 go accuracy exceeded 80% and no-go accuracy fell below 20% (*n*=1).
- non-responsiveness if Block 1 go accuracy fell below 20% and no-go accuracy exceeded 80%) (*n*=5). Additionally, data were excluded from block 2 only if block 2 go accuracy fell below 20% and no-go accuracy exceeded 80%)(*n*=3).

In instances of technical error (i.e. the touchscreen did not register the participant’s response, for example due to a very soft tap or the child having wet or sticky fingers), the actual response, as identified from the video, was used to compute the inhibitory control score. However, data were excluded if 6 or more trials were incorrectly registered in a block. In order to ensure that participants understood the task, data were excluded if participants completed fewer than 3 demo trials or fewer than 3 practice trials; see Supplementary Table 1.1. In order to ensure that the inhibitory demands of the task were as designed, data were excluded if participants completed fewer than 12 valid go trials or 4 valid no-go trials in a block. Videos were coded by an undergraduate student, blind to group status (CG). Videos for 11 participants (528 trials) were independently double-coded by a second researcher (AH). Inter-coder reliability was excellent for judgements of accuracy (Κ=.920, *p*<.001) and validity (Κ=.930, *p*<.001).

The dependent variable for each block was the d prime (d′) sensitivity index, a standardized difference between the hit rate (proportion of go trials to which there was a correct tap) and the false alarm rate (proportion of no-go trials to which there was an incorrect tap), calculated by subtracting the z-transformed FA rate from the z-transformed hit rate: i.e. d’prime = (z(Hit) minus z(FA)).

### Hide-and-Seek

The aim of this novel touchscreen task was to elicit individual differences in children’s ability to respond first by one rule, then by another (inhibiting incorrect responses linked to the previous rule(s). Participants were first asked “Which house is big/red/blue/yellow” (from a choice of two houses, equally matched on all but the salient property, and counter-balanced by side). The purpose of this was to check understanding of colour and size; if the participant did not pass the four concept-checking trials by touching the correct house on each trial, the task was still administered but the participant’s data was excluded (see Supplementary Table 1.1). A static image of the Sesame Street puppet Kermit was then shown in the centre of the screen and participants were told “This next game is a finding game. This is Kermit. Kermit likes to hide in the big house. Are you ready to find Kermit?” The experimenter then initiated the block by tapping the centre of the screen, then withdrew behind the child. In each block, when the correct house was tapped the image was replaced with a video of the relevant character dancing in the centre of the screen, for 3000ms, then the next trial began. If the incorrect house was tapped, the screen went blank for 3000ms, then the next trial began.

In block 1, the participant was presented with 2 houses on each trial, with the location of the correct house counterbalanced by side across the block. The first 4 trials in the block comprised black-and-white houses differing only in size (pre-shift with one dimension). For the next 4 trials, houses differed both in size and colour (pre-shift with two dimensions). At the end of block 2, a static image of the Sesame Street puppet Elmo was then shown in the centre of the screen and participants were told “This is Elmo. Elmo likes to hide in the red house. Are you ready to find Elmo?” Block 2 comprised 8 ‘conflict condition’ trials, each with 2 possible targets per trial, where the correct target (red house) was small (thus conflicting with the previous block) and 8 ‘distract condition’ trials, where the incorrect target (blue house) was also small so there was no conflict with the prior block. The location of the correct house was counterbalanced by side across the block. At the end of block 3, a static image of another puppet was shown in the centre of the screen and participants were told “This is Slinky. Slinky likes to hide in the yellow house. Are you ready to find Slinky?” Block 3 comprised 6 ‘double-conflict condition’ trials, where the correct target (small yellow house) was presented alongside a big red house (thus conflicting with both prior blocks) and a small blue house (distractor) and 6 ‘single-conflict’ trials where the correct target was presented alongside a small red house (thus conflicting just with the prior block) and small blue house (distractor). Targets were arranged at equidistant points from the centre of the screen, counter balanced by position across the block.

The dependent variables were the number of correct trials, as a proportion of all valid trials from the pre-shift with two dimensions block onwards (see Supplementary Table 1.1 for exclusions) As the prompt appeared on the screen until a response was registered, and all participants looked whilst giving a response, we did not need to exclude any trials as invalid due to inattention. In instances of technical error (i.e. the touchscreen did not register the child’s response, for example due to a very soft tap or the participant having wet or sticky fingers) the actual response (as identified from the video) was used to compute trial accuracy. Videos were coded by an undergraduate student, blind to group status (CG). Videos for 11 participants (528 trials) were independently double coded by a second researcher (AH). Inter-coder reliability was excellent (Κ=.920, p<.001).

### Delayed Alternation

This task was created as a touchscreen version of a working memory paradigm introduced by Espy, Kaufmann, McDiarmid, and Glisky (1999). The participant was shown an image on screen with 2 green doors positioned to the left and right of an image of a television and told “Now we’re going to play a finding game. Behind one of these doors is a funny man who wants to show us some cartoons… I think he’s over here”. During the instruction the experimenter moved her finger from pointing at the centre of the screen to the first door (always to the right hand side for the first demonstration trial) which she then tapped. There followed a reward sequence (described below), followed by three more demonstration-and-reward trials with the target alternating on each trial. After the fourth demonstration trial the experimenter said “Now it’s your turn, can you find the funny man?” If the participant correctly tapped the opposite door to the previous trial they were shown the reward sequence; a stop-motion animation of a figure coming out from the door and turning on a television which then displayed a short self-contained animated sequence. In total this animated sequence lasted for 7 seconds (2-year visit) or 10 seconds (3-year visit) and served both as a reward and to draw the participant’s eyes to the centre of the screen so that they would not use gaze to maintain a representation of which door to tap). If the response was incorrect the door ‘opened’ and was shown to be empty and then a blue screen was displayed. Again in total this distractor lasted for 7 or 10 seconds (2- and 3-year visits respectively). Throughout the task, the reward cartoon was only activated by tapping the location opposite from the child’s last correct response. Therefore, to achieve the maximal correct score, the participant had to alternate tapping between right and left doors on each successive trial; thus the task aims to elicit individual differences in the ability to hold in mind a rule (tap 1 of 2 alternating locations), update the representation of the correct response, and inhibit the previous response.

To minimize demands on participants, at the 2-year visit if an incorrect location was selected 11 times the task was terminated (the participant was shown a Finished screen and told ‘Well done, you’ve finished!’) (*n*=34). Additionally, if at any point the participant indicated that they wanted to stop (verbally or otherwise) the task was terminated. To avoid early termination of the task biasing the data, data were only included if the participant had completed more than five trials. The dependent variable was the number of correct retrievals as a percentage of the total trials administered (Espy et al., 2002).

Data were coded by 3 coders (CA, EC, RLP). 15 files (223 trials) were double-coded by EC and CA. Agreement of the accuracy of each trial was high: Κ=.928,*p*<.001. Nine files (127 trials trials) were double coded by EC and CA. Agreement was complete: Κ=1.0,*p*<.001.

## 1.4 Object-based tasks

For the object-based tasks, the participant was seated at a low table with the experimenter seated at 90^○^.

### Prohibition

Following the protocol described by Friedman et al. (2011), the participant was seated at a low table with the experimenter seated at 90^○^. The experimenter drew the participant’s attention to an attractive toy (a glitter wand), made eye contact with the participant, placed the toy on the table within reach of the participant and said “[Child’s name], don’t touch,” then moved away and pretended to be busy. The experimenter released the prohibition by saying “It’s okay, you can touch it now” after the participant touched the toy or after 30 seconds if the participant did not touch. The dependent variable was the latency to touch the toy in seconds, trimmed to a maximum of 30 (the point at which the prohibition was released). Researchers (MH, FH, AO, RLP, CG), blind to FH status, coded the tasks. Twenty-two files were double coded, with each coder double-coding a minimum of 6 files. Agreement between each pair of raters was excellent (r>. 89, r_s_>.88).

### Spin the Pots

Following Hughes and Ensor (2005), the participant was seated at a low table with the experimenter seated at 90^○^. On the table was placed a turntable, and on that 8 visually-distinct pots arranged in a ring. The participant was encouraged to observe the researcher hiding a ‘treasure’ (a gemstone-style sticker) in 6 of the pots (one treasure per pot). In each trial, the researcher covered the pots with a cloth, spun the turntable, then lifted the cloth and invited the participant to point to a pot with treasure in it. After a pot was selected the researcher opened the pot to reveal whether or not it had treasure inside: if so the treasure was given to the child and the lid replaced; if not the researcher commented that the pot was empty and replaced the lid. The turntable was then re-covered and a new trial begun. The dependent variable was 12 (the maximum number of searches) minus the number of errors made; see SM 1.1. for data cleaning protocol. The experimenter live scored the task (scores missing for n=14 due to data not being inputted). A researcher (CG) blind to group coded from video (scores missing for n=8 due to video not recording). Agreement for the 93 double coded files was excellent (r_s_=.975,p<.001). Discrepant scores (n=6) were reviewed by a second researcher (AH) and a final score allocated.

## 1.5 Data reduction: further detail

Prior to analysis, Hide-and-Seek task scores were log-transformed so that data approximated a normal distribution. All other task raw scores approximated a normal distribution with the exception of the Prohibition task which showed a bimodal distribution (toddlers either touching the toy within 5 seconds, or not at all). To preserve variance, we did not reduce scores to a binary score but adjusted model estimators to account for the data distribution. All scores were converted to *z*-scores to minimize the impact of different variable scaling on fitting model invariance. Prior to EFA, Bartlett’s test of sphericity was used to ensure that the correlation matrix was not random and the Kaiser-Meyer-Olkin statistic was required to be above a minimum of .50. Following best-practice guidance for EFA (Watkins, 2018), our decision of the number of factors to accept was influenced by 2 criteria, established a priori:

1. Visual examination of a scree plot using parallel analysis to statistically simulate 100 sets of random data with the same number of variables and participants as the real data. The eigenvalues for the 100 sets were averaged and compared with the components extracted from the real data. The eigenvalues for FA extracted from real data that exceed those extracted from random data for FA indicate the number of factors to retain.
2. Parsimony and factor adequacy: The model with the fewest number of factors is preferred. Given the number of participants in this study, and modest associations typically found in toddler research, pattern coefficients >.25 are considered salient (i.e., both practically and statistically significant). Complex loadings (i.e., salient on more than one factor) should be rejected to honor simple structure. Factors with a minimum of three salient pattern coefficients, and that are theoretically meaningful are considered adequate.

Due to the nature of the constructs, it was assumed that factors would be correlated. Therefore, an oblimin rotation was employed. A Mardia test, performed using the QuantPsyc package (Fletcher & Fletcher, 2010) indicated significant Kurtosis (*p*=.04). Therefore the OLS estimator was used. Factor scores were computed using the Bartlett method with mean imputation of missing items, then taken forward for simple linear regression analyses to test for predictive associations between 2- and 3-year scores. A Benjamini-Hochberg correction was applied to correct for the false discovery rate within each family-wise test.

EFA was conducted with the psych package (Version 2.3.3) (Revelle & Revelle, 2015) of R (Version 4.2.1). All other analysis and data preparation was performed in SPSS (Version 28.0.0.0).

### Missing data

The proportion of missing data ranged from 37% (Reversal learning) to 16% (Delayed Alternation) at the 2-year visit, and 48% (Go/No-Go block 2) to 3% (Prohibition task) at the 3-year visit, which is within the range of reported proportions of missing data in EF studies with typically-developing 2- to 6-year-olds (Wiebe et al. 2008). Logistic regression analyses were conducted to assess relations between missingness and age, and FH group for each task. Neither of these variables were significantly associated with missing data on any of the tasks at either timepoint; therefore all complete cases were analyzed

# Supplementary Materials 2: EF scores and data reduction

## 2.1 Task level scores at the 2- and 3-year visits

Supplementary Table 2.1.1 Task-level EF scores at 2 and 3 years

|  |  | **No-FH-autism/ADHD** | **FH-autism/ADHD** |
| --- | --- | --- | --- |
| **2 years** |  |  |  |
| Anti-saccade | Mean (SD) | .56 (.20) | .50 (.31) |
|  | *n* | 20 | 80 |
| Reversal Learning | Mean (SD) | .75 (.19) | .68 (.25) |
|  | *n* | 12 | 66 |
| Hide the Toy | Mean (SD) | .52 (.20) | .47 (.18) |
|  | *n* | 21 | 79 |
| Prohibition | Mean (SD) | 20.76 (12.45) | 13.82 (13.50) |
|  | *n* | 21 | 79 |
| Delayed alternation | Mean (SD) | .39 (.17) | .41 (.22) |
|  | *n* | 22 | 84 |
| **3 years** |  |  |  |
| Anti-saccade | Mean (SD) | .48 (.21) | .40 (.32) |
|  | *n* | 17 | 70 |
| Reversal Learning | Mean (SD) | .74 (.20) | .72 (.21) |
|  | *n* | 18 | 80 |
| Prohibition | Mean (SD) | 28.70 (5.58) | 24.38 (9.93) |
|  | *n* | 20 | 99 |
| Go/No go block 1 | Mean (SD) | -.13 (.54) | .01 (.94) |
|  | *n* | 13 | 67 |
| Go/No go block 2 | Mean (SD) | -.16 (.85) | -.14 (.97) |
|  | *n* | 12 | 49 |
| Hide and Seek | Mean (SD) | .83 (.24) | .76 (.21) |
|  | *n* | 13 | 57 |
| Delayed alternation | Mean (SD) | .39 (.17) | .41 (.22) |
|  | *n* | 22 | 84 |

## 2.2 Sex differences in EF scores at the 2- and 3-year visits

Supplementary Table 2.2.1 Sex differences in EF scores at 2 and 3 years

|  |  | **Boys** | **Girls** | **Test statistic** | ***p*** |
| --- | --- | --- | --- | --- | --- |
| **2 years** |  |  |  |  |  |
| Executive Attention | Mean (SD) | .07 (.85) | -.11 (.91) | *t* = 1.086 | .230 |
|  | *n* | 59 | 47 |  |  |
| Hide the Toy | Mean (SD) | .49 (.18) | .48 (.19) | *t* = 0.231 | .817 |
|  | *n* | 52 | 48 |  |  |
| Prohibition | Mean rank | 46.58 | 54.75 | *U* = 1044.000 | .141 |
|  | *n* | 52 | 48 |  |  |
| Delayed alternation | Mean rank | 54.52 | 52.40 | *U*=1346.500 | .723 |
|  | *n* | 55 | 51 |  |  |
| **3 years** |  |  |  |  |  |
| Simple EF | Mean (SD) | -.057 | .045 | *t* = -0.563 | .574 |
|  | *n* | 66 | 57 |  |  |
| Complex EF | Mean (SD) | .007 | -.006 | *t* =-.071 | .944 |
|  | *n* | 66 | 57 |  |  |

## 2.3 EFA: 3-year visit

The Scree plot produced using parallel analysis, indicated that a 2- or 3-factor model should be selected (on the basis that the eigenvalue from the observed data is larger than the 95th percentile of the distribution of null factor eigenvalues generated from the synthetic data)^1,2^; see Supplementary Figure 2.1. Factor loadings for the 2- and 3-factor models are shown in Supplementary Table 2.2.1. The 3-factor model was rejected (as per a priori selection criteria) as Factor 1 had fewer than three salient pattern coefficients, and showed complex loadings.


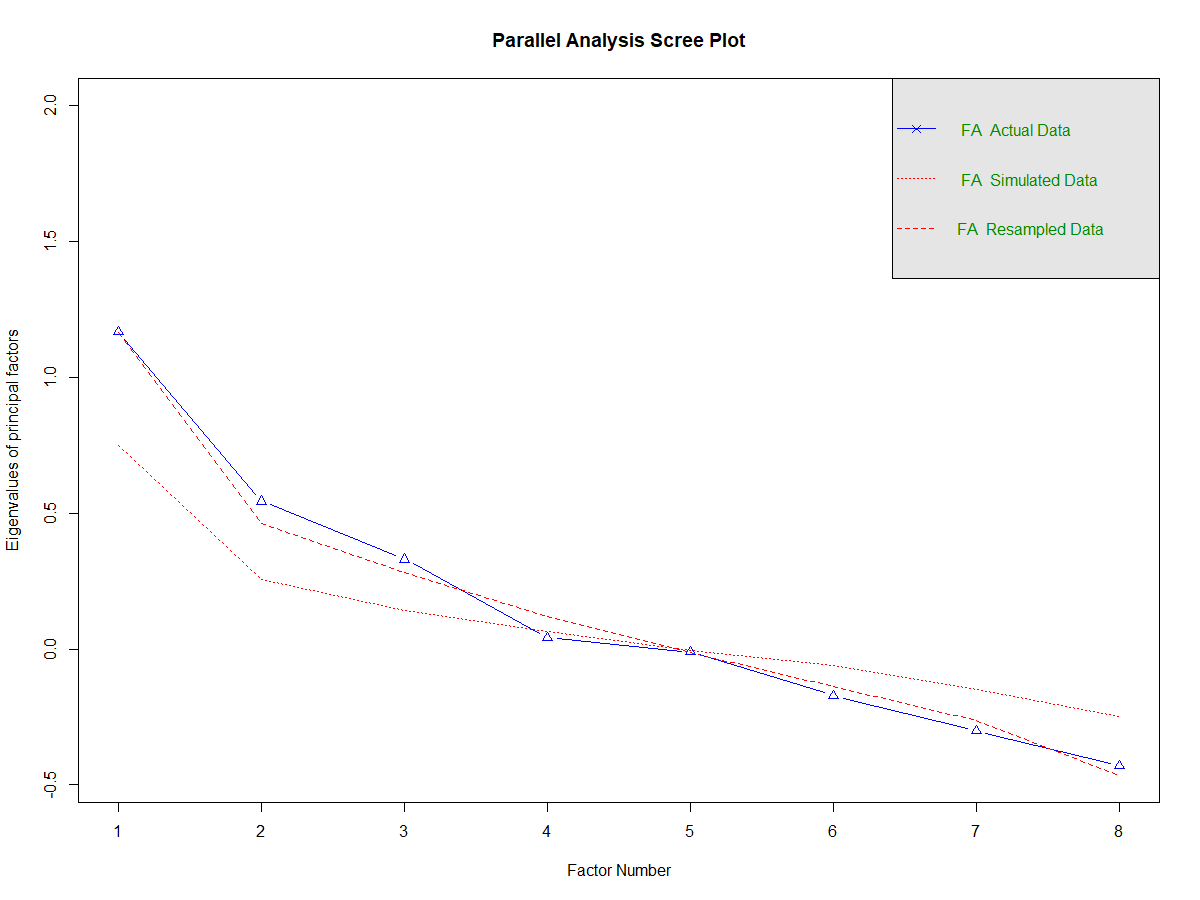


Supplementary Figure 2.3.1. Scree Plot

In the 2-factor model, Factor 1 accounted for 13% of the total variance (51% of the variance explained by the model). Factor 2 accounted for a further 13% of the total variance (49% of the proportion explained).

Supplementary Table 2.3.1. Factor loadings for 2- and 3-factor models using 36-month data, using the pattern matrix

| **2 factor model** | Factor 1 | Factor 2 |  | *h*^2^ |
| --- | --- | --- | --- | --- |
| Antisaccade | 0.11 | **0.37** |  | 0.15 |
| Reversal Learning | -0.17 | **0.63** |  | 0.42 |
| Prohibition | **0.53** | -0.02 |  | 0.28 |
| Go/No-Go block 1 | 0.22 | **0.37** |  | 0.19 |
| Go/No-Go block 2 | 0.25 | **0.42** |  | 0.24 |
| Hide-and-Seek | **0.29** | 0.19 |  | 0.12 |
| Delayed Alternation | **0.73** | -0.02 |  | 0.54 |
| Spin the Pots | 0.15 | **0.35** |  | 0.15 |
|  |  |  |  |  |
| **3 factor model** | Factor 1: | Factor 2 | Factor 3: | *h*^2^ |
| Antisaccade eyetracking | 0.20 | **0.46** | -0.07 | 0.24 |
| Reversal learning eyetracking | -0.13 | **0.64** | 0.01 | 0.43 |
| Prohibition task (Glitter Wand) | **0.85** | 0.00 | -0.04 | 0.72 |
| Go/No-Go task Block 1 | 0.22 | **0.36** | 0.09 | 0.20 |
| Go/No-Go task Block 2 | 0.03 | **0.26** | **0.48** | 0.34 |
| Hide and Seek task | -0.03 | -0.05 | **0.71** | 0.49 |

*h*^2^ **^=^** communality. Salient pattern coefficients >.25 in boldface.

Results using Varimax rotation were almost identical, as would be expected given correlations between Factor 1 and 2 of .02.

# References

1 Horn, J. L. A rationale and test for the number of factors in factor analysis. *Psychometrika* **30**, 179-185 (1965).

2 Finch, W. H. Using fit statistic differences to determine the optimal number of factors to retain in an exploratory factor analysis. *Educational and psychological measurement* **80**, 217-241 (2020).
